# FYN regulates cell adhesion at the blood-testis barrier and the apical ectoplasmic specialization *via* its effect on Arp3 in the mouse testis

**DOI:** 10.3389/fimmu.2022.915274

**Published:** 2022-08-09

**Authors:** Yue Yang, Mingxia Yao, Jie Zeng, Dongwang Zheng, Qin Li, Ya Ni, Xiang Xiao

**Affiliations:** ^1^ Center for Reproductive Health, School of Pharmaceutical Sciences, Hangzhou Medical College (Zhejiang Academy of Medical Sciences), Hangzhou, China; ^2^ School of Pharmaceutical Sciences, Hangzhou Medical College, Hangzhou, China; ^3^ Zhejiang Provincial Laboratory of Experimental Animal’s & Nonclinical Laboratory Studies, Hangzhou Medical College, Hangzhou, China

**Keywords:** testis, Sertoli cells, spermatogenesis, blood-testis barrier (BTB), cytoskeleton, non-receptor tyrosine kinase (nRTK), FYN, Arp3

## Abstract

FYN is a non-receptor tyrosine kinase of the SRC family that facilitates virus entry across epithelial tight junctions. However, the role of FYN in mammalian testes in maintaining the blood-testis barrier (BTB) integrity and the adhesion of germ cells to Sertoli cells are not well defined. Here, we show that FYN is a component of the BTB and the apical ectoplasmic specialization (ES) at Sertoli-Sertoli and Sertoli-spermatid interfaces, respectively, and is expressed extensively in mouse testes during postnatal development. FYN was shown to be structurally linked to the actin and microtubule-based cytoskeletons. An *in vivo* model was used to explore the modulatory effect of FYN on BTB and apical ES dynamics within the testes when adult mice were treated intraperitoneally with CdCl_2_ (3 mg/kg body weight). The CdCl_2_-induced epithelial restructuring was associated with a transient increase in the interaction between FYN and the actin branching/nucleation protein Arp3, as well as an induction of Arp3 phosphorylation, which possibly lead to actin cytoskeleton remodeling, resulting in BTB damage and germ cell loss in the seminiferous epithelium. Based on the results, we propose a model in which FYN and Arp3 form a protein complex that is responsible for junction reorganization events at the apical ES and the BTB. It is also possible for viruses to break through the BTB and enter the immunoprivileged testicular microenvironment *via* this mechanism.

## Introduction

A key component of immune privilege in mammalian testes is the blood-testis barrier (BTB), a unique structure within the seminiferous epithelium for maintaining spermatogenesis (1). The BTB is formed by junctions between adjacent Sertoli cells near the basement membrane and segregates the seminiferous epithelium into two compartments: basal and apical (adluminal). As a physical and immunological barrier in the seminiferous tubule that controls molecules and cells from entering or leaving the lumen, the BTB creates a specialized microenvironment that isolates postmeiotic germ cell development in the apical compartment from the systemic circulation. In contrast to blood-tissue barriers in other organs, such as the blood-epididymis, blood-brain, and blood-eye barriers, where tight junctions (TJ) rest on the apex of the cell epithelium and/or endothelium followed by the adherens junction (AJ), the BTB is composed of coexisting actin-based TJ, basal ectoplasmic specialization (ES), and gap junctions, along with intermediate filament-based desmosomes (2–4). The ES is an atypical AJ type that is unique to the testis. It features densely packed bundles of actin filaments sandwiched between the plasma membrane of adjoining Sertoli cells and cisternae of the endoplasmic reticulum at the basal ES. Furthermore, an additional ES, known as the apical ES, lies between elongating/elongated spermatids and Sertoli cells, which allows for proper orientation of spermatids as they develop while maintaining their attachment to the Sertoli cells (4–6). The ES at the BTB and at the apical ES is highly dynamic. During the late stage VIII of the seminiferous epithelial cycle in adult rodent testes, the BTB has to undergo extensive restructuring, so that preleptotene spermatocytes are able to pass through the barrier during their differentiation into leptotene and zygotene spermatocytes. It is vital, though, that the integrity of the BTB be preserved so that the microenvironment behind the barrier can remain undisturbed and postmeiotic germ cell antigens can be protected from the immune system (2, 7). Although molecular mechanisms governing BTB restructuring during spermatocyte transit remain poorly understood, growing evidence suggests a “new” barrier is formed behind transiting preleptotene spermatocytes, with the “old” barrier disassembled above them to allow spermatocyte passage while maintaining the barrier integrity (2, 7, 8). The apical ES, on the other hand, is disassembled to facilitate fully developed elongated spermatids (spermatozoa) to enter the tubule lumen at spermiation, which corresponds to the time when the BTB is transiently “opened” or reorganized. It is noteworthy that damage to the BTB results in secondary disruption to the apical ES, and vice versa (2, 4–6). In response to these interconnected and massive junctional remodeling processes, actin filament bundles located at the basal and apical ES are periodically converted into highly branched networks, involving a complex set of signaling molecules and a rapid turnover of cell junctions (2, 6, 9). In previous studies, it has been shown that the distribution and/or recruitment of the ES-associated proteins to the BTB and apical ES is controlled, at least in part, by protein phosphorylation events that target tyrosine, serine, and/or threonine residues on proteins (10–13).

We have previously shown that SRC and YES, two non-receptor tyrosine kinases belonging to the SRC family of kinases (SFKs) with different palmitoylation states in their SH4 domain (14, 15), function divergently and share roles in intracellular protein trafficking events in Sertoli cells to maintain protein turnover and homeostasis at the BTB and apical ES (12, 13). Specifically, non-palmitoylated SRC transported rapidly between the plasma membrane and late endosomes or lysosomes (14, 15) likely participates in endosome-mediated protein degradation at the “old” BTB and in the apical ES disassembly, as well as in Sertoli cell phagocytosis of the residual bodies (the residual cytoplasm shed by mature sperm). Mono-palmitoylated YES, preferentially redirected by Rab11 from the Golgi pool of caveolin to the plasma membrane (15), is essential for endocytosis, recycling and/or transcytosis of integral membrane proteins at the basal and apical ES in rat testes (12, 16). FYN, which is dually palmitoylated, is one of three widely expressed members of the SFK family after SRC and YES. However, its role in seminiferous epithelial restructuring is not as well understood. FYN has been observed to be predominantly localized at the basal and apical ES in mouse testes and to be a regulatory protein embedded in cytoskeletal structures rich in filamentous (F-) actin and modulating ES integrity by contributing high levels of protein tyrosine phosphorylation at the site (17). In rat testes, FYN has been detected to phosphorylate and stimulate plakoglobin (an ES/desmosome protein) interaction with α-catenin at the BTB (18). We sought to further elucidate FYN’s role distinct from SRC or YES in the basal ES/BTB and apical ES, utilizing an *in vivo* cadmium model that induces large-scale epithelial remodeling in the mouse testes. Here, we report the presence of protein complexes consisting of FYN and classic actin regulatory proteins such as Arp3 (actin-related protein 3), Eps8 (epidermal growth factor receptor pathway substrate 8), and annexin A2 at the TJ and ES, describe how FYN modulates BTB and apical ES dynamics through interactions with Arp3, and present a hypothetical working model. There has been experimental evidence that viruses use SFKs for endothelial/epithelial cell, vascular, and tissue penetration (19–23). According to our findings, FYN and Arp3 may work together to breach the BTB, and viruses may use similarly optimized strategies to disassemble TJ and/or AJ at the BTB to invade the immunoprivileged environment of the testis.

## Materials and methods

### Animals

Male Crl: CD1(ICR) mice (aged 2–10 weeks) were purchased from Shanghai Silaike Experiment Animal Co., Ltd (Shanghai, China) and housed at the Zhejiang Laboratory Animal Center [animal use license number: SYXK (zhe) 2019-0011]. The animals were maintained in a temperature- and humidity-controlled environment with 12:12 hour light-dark cycles and free access to food and water. The mice were treated humanely in accordance with the guidelines of the Institutional Animal Care and Use Committee of Hangzhou Medical College (Zhejiang Academy of Medical Sciences). Animal carcasses were collected and disposed of by Hangzhou Dadi Weikang Medical Environmental Protection Co., Ltd.

### Antibodies

The primary and secondary antibodies used in this study are listed in **Table 1**. For each antibody, the name, host animal species, supplier, catalog number, and dilution used in each application are all stated. The working concentrations of each antibody were optimized based on the manufacturer’s recommended dilutions and experimental settings. Different FYN antibodies yielded consistent results in different applications.

**Table 1:** Antibodies used in this study.

Antibody (RRID)	Host	Vendor	Catalog No.	Application(s)/Dilution(s)
**FYN (AB_631528)**	Rabbit	Santa Cruz Biotechnology	sc-16	IB (1:400), IHC (1:100), IF (1:100)
**FYN (AB_11011279)**	Rabbit	Novus Biologicals	NBP1-82685	IB (1:500), IF (1:100)
**FYN**	Rabbit	Abcam	ab184276	IB (1:1000), IP (2 μg)
**Occludin (AB_88065)**	Rabbit	Thermo Fisher Scientific	71-1500	IB (1:250)
**Occludin (AB_2533101)**	Mouse	Thermo Fisher Scientific	33-1500	IB (1:250), IF(1:100)
**Occludin (AB_653540)**	Rabbit	Santa Cruz Biotechnology	sc-5562	IP (2 μg)
**CAR (AB_2087557)**	Rabbit	Santa Cruz Biotechnology	sc-15405	IB (1:200)
**N-Cadherin (AB_647794)**	Rabbit	Santa Cruz Biotechnology	sc-7939	IB (1:200)
**β-Catenin (AB_138792)**	Mouse	Thermo Fisher Scientific	13-8400	IB (1:250), IF (1:100)
**Nectin-3 (AB_2284699)**	Rabbit	Santa Cruz Biotechnology	sc-28637	IB (1:200)
**SRC (AB_2302631)**	Mouse	Millipore	05-184	IB (1:500)
**YES (AB_397758)**	Mouse	BD Biosciences	610375	IB (1:500)
**β-Actin (AB_476697)**	Mouse	Sigma-Aldrich	A2228	IB (1:2000)
**α-Tubulin (AB_2241126)**	Mouse	Abcam	ab7291	IB (1:5000)
**β-Tubulin (AB_2210370)**	Rabbit	Abcam	ab6046	IB (1:4000)
**Arp3 (AB_476749)**	Mouse	Sigma-Aldrich	A5979	IB (1:3000)
**Eps8 (AB_397544)**	Mouse	BD Biosciences	610143	IB (1:4000)
**Annexin A2 (AB_2057311)**	Rabbit	Proteintech	11256-1-AP	IB (1:2000)
**Annexin A2 (AB_397479)**	Mouse	BD Biosciences	610068	IB (1:2000)
**GAPDH (AB_2107448)**	Mouse	Abcam	ab8245	IB (1:1000)
**ZO-1 (AB_87181)**	Mouse	Thermo Fisher Scientific	33-9100	IF (1:100)
**Phosphotyrosine (AB_2533927)**	Rabbit	Thermo Fisher Scientific	61-5800	IP (2 μg)
**m-IgGκ BP-HRP (AB_2687626)**	Mouse	Santa Cruz Biotechnology	sc-516102	IB (1:3000)
**Goat anti-Rabbit IgG (H+L) Secondary Antibody (AB_2533967)**	Goat	Invitrogen	65-6120	IB (1:20000)
**Alexa Fluor 488 donkey anti-Mouse IgG (H+L) (AB_141607)**	Donkey	Invitrogen	A-21202	IF (1:250)
**Alexa Fluor 555 donkey anti-Rabbit IgG (H+L) (AB_162543)**	Donkey	Invitrogen	A-31572	IF (1:250)
**Alexa Fluor 488 Goat anti-Rabbit IgG (H+L) (AB_2576217)**	Goat	Invitrogen	A-11034	IF (1:250)

RRID, Research Resource Identifier; IB, immunoblotting; IHC, immunohistochemistry; IF, immunofluorescence; IP, immunoprecipitation; m-IgGκ BP, Mouse IgGκ light chain binding protein; HRP, horseradish peroxidase; IgG (H+L), Immunoglobulin G heavy and light chain.

### Treatment of mice with CdCl_2_

CdCl_2_ (Sigma-Aldrich, Shanghai, China) dissolved in sterile saline [0.89% NaCl (w/v)] was given intraperitoneally (i.p.) to male ICR mice (8-week old) at a single dose of 3 mg/kg body weight (b.w.) to induce disruption of cell junctions between Sertoli-Sertoli and Sertoli-germ cells, and thus the exfoliation of germ cells from the seminiferous epithelium (24–27). Mice (n=7-10 mice per time point) were killed by CO_2_ asphyxiation at different time points (6, 16, 24, and 48 hours after CdCl_2_ treatment). The control group (time point 0 hour, n=10 mice) were left untreated (24, 25).

### Immunohistochemistry (IHC), immunofluorescence (IF) and histological analysis

Freshly isolated mouse testes were immersed in Bouin’s fixative overnight at 4°C, followed by ethanol dehydration and xylene clearing steps (28). Following that, tissue samples were embedded in paraffin, and 5-μm-thick sections were cut from the paraffin blocks and placed on positively charged adhesive glass slides (CITOGLAS World Thai, Haimen, China), which were then baked overnight at 37°C. After that, sections were deparaffinized in xylene and rehydrated through graded concentrations of ethanol in water until pure water was reached. For IHC experiment, a two-step plus^®^ Poly-HRP Anti-Mouse/Rabbit IgG Detection System (PV-9000, ZSGB-BIO, Beijing, China) was used to stain tissue sections with an anti-FYN antibody (**Table 1**). Immunohistochemical signals were generated using a DAB (3,3’-diaminobenzidine) substrate kit (ZLI-9017, ZSGB-BIO). Sections were counterstained with hematoxylin and mounted with neutral balsam (Shanghai Specimen and Model Factory, China). For dual-labeled IF microscopy, paraffin or frozen sections were used. Fresh testes were embedded in Tissue-Tek^®^ O.C.T. Compound (Sakura Finetek USA), snap-frozen in liquid nitrogen, and sliced at 7 μm using a Leica CM1950 cryostat for frozen sections. After incubation with specific primary antibodies, sections were treated with Alexa Fluor secondary antibodies (**Table 1**) before being mounted with DAPI (Thermo Fisher Scientific, Waltham, USA) and viewed under a Nikon Eclipse 80i microscope (Tokyo, Japan). Images were captured using an Imaging Software NIS-Elements F Ver5.21.00 (Nikon Instruments Inc., Melville, USA) (29). For protein colocalization analysis, image overlays were produced with Adobe Photoshop (Adobe Systems Inc., San Jose, USA).

In order to evaluate the morphological and physiological changes in mouse testes after CdCl_2_ administration, paraffin-embedded sections from the 0- (control) and 16-hour post-treatment groups were stained with hematoxylin and eosin (H&E) as previously described (25, 30). Briefly, the deparaffinized and rehydrated sections were sequentially submerged in Carazzi’s hematoxylin solution [10 mM hematoxylin (C_16_H_14_O_6_), 0.105 M AlK(SO_4_)_2_•12H_2_O, 3 mM NaIO_3_, and 20% glycerol (v/v)], acid-alcohol decolorizer [1% HCl in 75% ethanol (v/v)], bluing solution [0.2% NH3•H2O (v/v)], and eventually 0.5% eosin Y (w/v) alcoholic solution for cytoplasmic counterstain. The sections were dehydrated and sealed with neutral balsam (G8590, Solarbio, China). To visualize the alterations in F-actin organization in the testes, 7-µm frozen sections were fixed with 4% paraformaldehyde (w/v), permeabilized with 0.1% Triton-X100 (v/v), blocked with 1% BSA (w/v) in PBS, and incubated with rhodamine phalloidin (R415, Thermo Fisher Scientific, 1:50 dilution in PBS). The images were acquired and processed as described above.

### Immunoblotting

Following CO_2_ euthanization of mice, testes were removed, immediately snap-frozen, and stored at -80°C for further use. Protein extracts from mouse testes were prepared in IP lysis buffer (P0013, Beyotime, Shanghai, China) freshly supplemented with 1 mM PMSF (ST506, Beyotime). The samples were sonicated and centrifuged at 15,000 g for 1 hour at 4°C, the supernatant was collected, and the total protein concentration was determined by using the BCA Protein Assay Kit (P0012, Beyotime) *via* the Cytation 3 Cell Imaging Multi-Mode Reader (BioTek, Winooski, USA). Approximately 30-50 µg of protein from testis lysates collected at different ages of mice or time intervals after CdCl_2_ administration was separated by SDS-PAGE, and then transferred onto PVDF membrane (EMD Millipore, Bedford, USA) for immunoblotting with specific antibodies (**Table 1**) as described previously (25). Bands of immunoreactive proteins were visualized by an ECL western blotting detection kit (P0018A, Beyotime) and captured with an Amersham Imager 600 (GE Healthcare Life Sciences, USA).

### Co-immunoprecipitation (Co-IP)

The mouse testis lysates were precleared using Protein A/G plus-agarose (Santa Cruz Biotechnology, Santa Cruz, USA), and approximately 800 µg of total protein from each sample was incubated with 2 µg of the corresponding antibody (**Table 1**) overnight at 4°C on a rotator. In the negative control, the antibody was substituted with normal rabbit IgG. Following a 6-hour incubation with 20 µl Protein A/G plus-agarose, the immunoprecipitated antigen-antibody complex was washed 4-5 times with IP lysis buffer, collected by centrifugation at 1,000 g for 5 min at 4°C, and subjected to immunoblotting analysis by using specific antibodies (**Table 1**) as previously described (25).

### Statistical analyses

Each experiment was repeated at least three times, with n=7-10 mice at each time point. Graph plotting and statistical analyses were performed with GraphPad Prism 6 (GraphPad Software, San Diego, CA, USA) and data were presented as the mean ± standard deviation (S.D.). One-way ANOVA followed by Dunnett’s test was used to compare the means of multiple experimental groups. P < 0.05 was considered statistically significant.

## Results

### FYN is highly expressed throughout the seminiferous epithelial cycle in the adult testis

**Figure 1:**
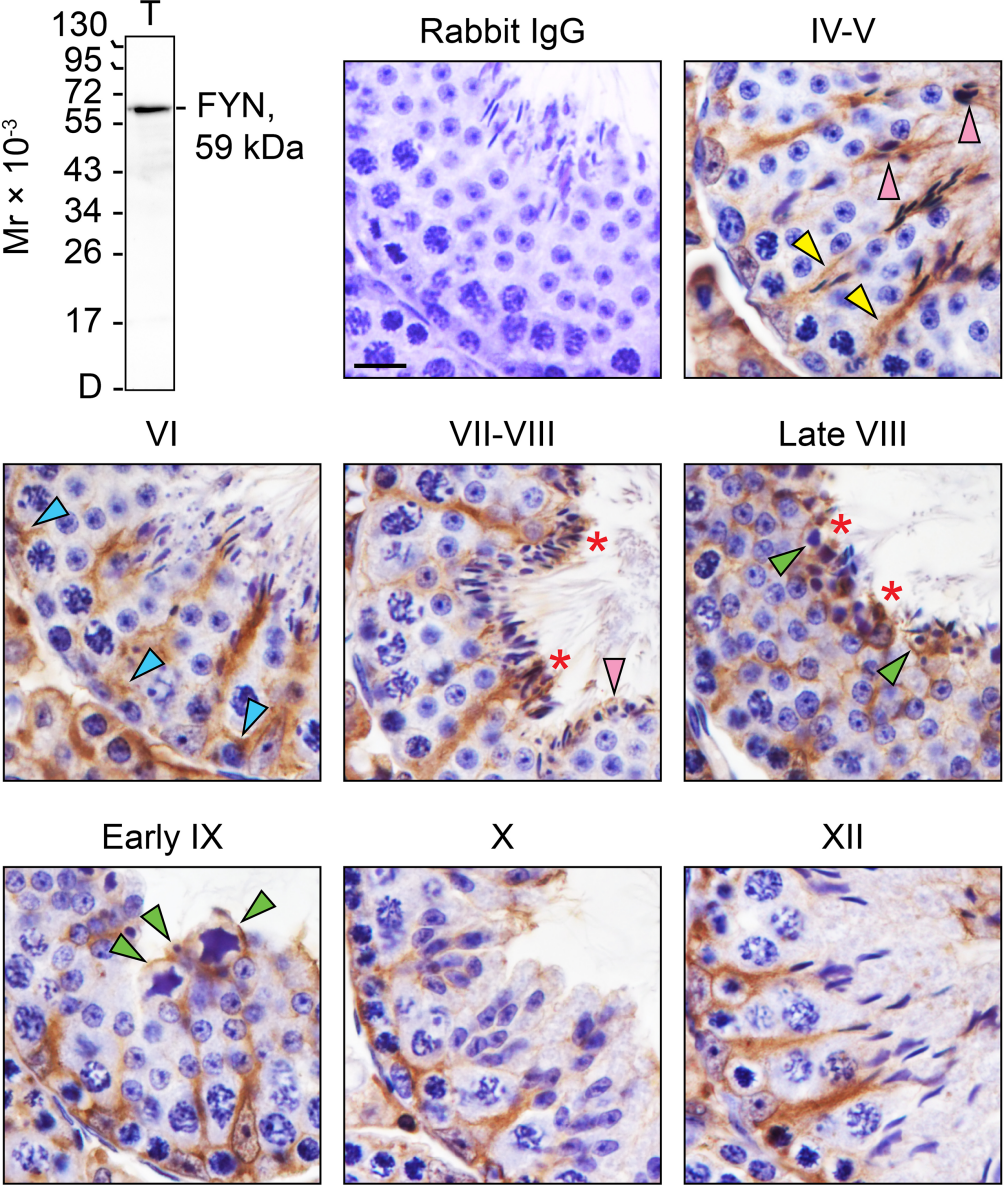
FYN expression and distribution patterns in adult mouse testes. Testes from 8-week-old mice were fixed in Bouin’s solution, paraffin embedded, and tissue sections were cut at 5 µm and immunostained using a rabbit anti-FYN polyclonal antibody (see **Table 1**). Seminiferous tubules from various stages of the seminiferous epithelial cycle (denoted by Roman numerals IV-XII) were displayed. FYN was found at the Sertoli cell stalk (yellow arrowheads), the elongating and elongated spermatids (pink arrowheads), and the blood-testis barrier (blue arrowheads) as a brown precipitate utilizing 3,3’-diaminobenzidine (DAB) enzymatic detection. FYN was also detected at the luminal edge (red asterisks), and around residual bodies/phagosomes (green arrowheads). No immunoreactive signal was present in the negative control when the primary antibody was replaced with rabbit IgG. Scale bar, 10 μm. This antibody used for IHC was specific for FYN when assessed by immunoblotting using lysate (30 µg of total protein) from mouse testes (T). Mr, molecular weight; D, dye front.

By IHC analysis, cellular expression and localization pattern of FYN in adult mouse testes (**Figure 1**, IV-XII) were revealed as compared to control (**Figure 1**, rabbit IgG). The monospecificity of the anti-FYN antibody used for IHC (**Table 1**) was demonstrated by immunoblotting (**Figure 1**, upper left), in which FYN arose as a single, prominent 59-kDa protein band by performing SDS-PAGE on testis lysate as previously reported (31, 32). The strong immunoreactivity of FYN was evident at all stages of the seminiferous epithelial cycle, extending from the basal lamina toward the tubule lumen along the tree-like stalks of Sertoli cell cytoplasm enclosing developing germ cells. A significant amount of immunoreactive FYN was also found around the heads of elongating/elongated spermatids and in the basal third of the seminiferous epithelium, which is in line with prior findings that FYN is localized at the apical ES and the BTB/basal ES (4, 12, 17, 18). As spermiation progressed from stage VII to late stage VIII, as well as after sperm release in stage IX, discrete FYN staining was observed at the tubule lumen in association with residual bodies and spermatozoa being discharged (**Figure 1**) (33).

### Postnatal expression of FYN parallels that of the BTB and the apical ES proteins

**Figure 2:**
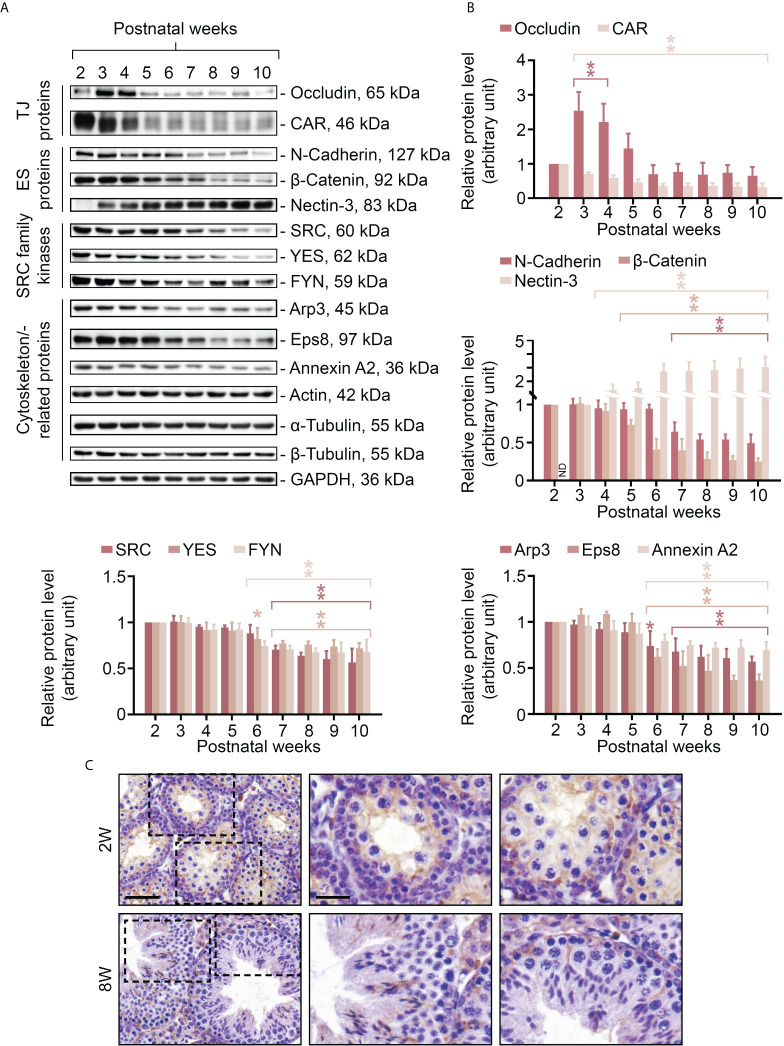
Changes in expression levels of FYN and BTB/apical ES-associated proteins in mouse testes during postnatal development. Male mice at 2-10 weeks old were terminated to obtain lysates of testes for immunoblotting experiments **(A)**. The steady-state levels of TJ proteins (occludin and CAR), ES proteins (N-cadherin, β-catenin and nectin-3), SRC family kinases (including FYN and another two family members SRC and YES, which are known to regulate BTB and apical ES), and cytoskeletal proteins (including actin and its binding proteins Arp3, Eps8, and annexin A2, as well as α- and β-tubulin) were examined. GAPDH was used as an internal control to ensure the equal protein loading across the gel (~50 µg protein/lane). Histograms **(B)** summarizing the results of immunoblot analysis in **(A)**. Each data point was first normalized against its corresponding GAPDH result, then against that of “postnatal week 2” (except for nectin-3, for which “postnatal week 3” was used), which was arbitrarily set to 1. Each bar = mean ± S.D. of three independent experiments. *P<0.05; **P<0.01 (Student’s t-test). Because the levels of actin, α- or β-tubulin stay constant during the postnatal testis development, the histogram for these proteins is excluded. IHC staining was used to examine the expression and localization pattern of FYN in testes in pups (2W, 2 weeks old) versus adults (8W, 8 weeks old) **(C)**. 5 μm-thick paraffin sections were immunostained using a rabbit anti-FYN polyclonal antibody (**Table 1**). Boxed areas with a dotted outline in the first column (2W versus 8W) are enlarged and presented to the right in the second and third columns, respectively. Scale bar, 45 μm (first column) and 20 μm (second column; also applies to the third column).

In order to obtain further insight into the relationship between FYN and cell adhesion within the seminiferous epithelium, and also to take advantage of the developmental timeline of testis maturation and the establishment of Sertoli/germ cell adhesion, the steady-state levels of several BTB/apical ES-associated proteins were assessed in testes ranging from 2 to 10 weeks of age by immunoblotting, including TJ proteins (occludin and CAR), ES proteins (N-cadherin, β-catenin, and nectin-3), SRC family kinases (including FYN and two other family members, SRC and YES, that are known to modulate BTB and apical ES dynamics) (12, 13), and cytoskeletal proteins (including actin and its binding proteins Arp3, Eps8 and annexin A2, as well as α- and β-tubulin) (**Figures 2A, B**). The proteins revealed by specific antibodies (**Table 1**), with the exception of FYN, are all well characterized and established junctional components at the BTB and germ cell adhesion (2, 34–36), and were thus appropriate as reference proteins for the study to understand FYN function. Junctional proteins were primarily detectable in postnatal testes 2 weeks after birth, with a downward trend beginning at 6-7 weeks when full fertility is reached (37). It may be that with aging and the cessation of new BTB assemblies, BTB dynamics decrease, and protein turnover remains at a minimum level sufficient for normal spermatogenesis, as observed with CAR whose steady-state level significantly declines during the maturation of testis and other organs (38). On the other hand, nectin-3, an apical ES integral membrane protein that is expressed exclusively by elongating/elongated spermatids (39, 40), did not appear until the third postnatal week, when the apical ES is expected to arise in step 8 spermatids at 24-25 days postpartum in mice (41, 42). The steady-state level of occludin, a TJ integral membrane protein, showed a transient surge around postnatal weeks 3-4, suggesting extensive *de novo* BTB formation. This observation is consistent with previous studies that the launching of a fully functional BTB takes longer than 3 weeks, even though the BTB begins to form in rodents before this age, as discussed in the literature (2, 43, 44). Throughout the postnatal testis development, the levels of actin, α- and β-tubulin remained constant (**Figure 2A**, histogram not shown). IHC analysis was conducted to compare the distribution of FYN in immature (**Figure 2C**, 2W) versus adult (**Figure 2C**, 8W) testes, and the results confirmed those in **Figure 2A** that when complete spermatogenesis and a functional BTB were established, FYN was less expressed in adults than in prepubertal mice. FYN immunostain diffusely packed the tubule by postnatal 2 weeks, while a stronger belt-like immunoreactive signal was noted at the base of the seminiferous epithelium (**Figure 2C**, 2W, magnified views), which corresponded to the location of the emerging BTB (43). The expression level of FYN remained relatively stable after 6 weeks postpartum, demonstrating that it is essential not only for the assembly, but also for the maintenance of the BTB and germ cell adhesion in adult mouse testes.

### FYN is an integral part of the adhesion protein complexes at the BTB and the apical ES

**Figure 3:**
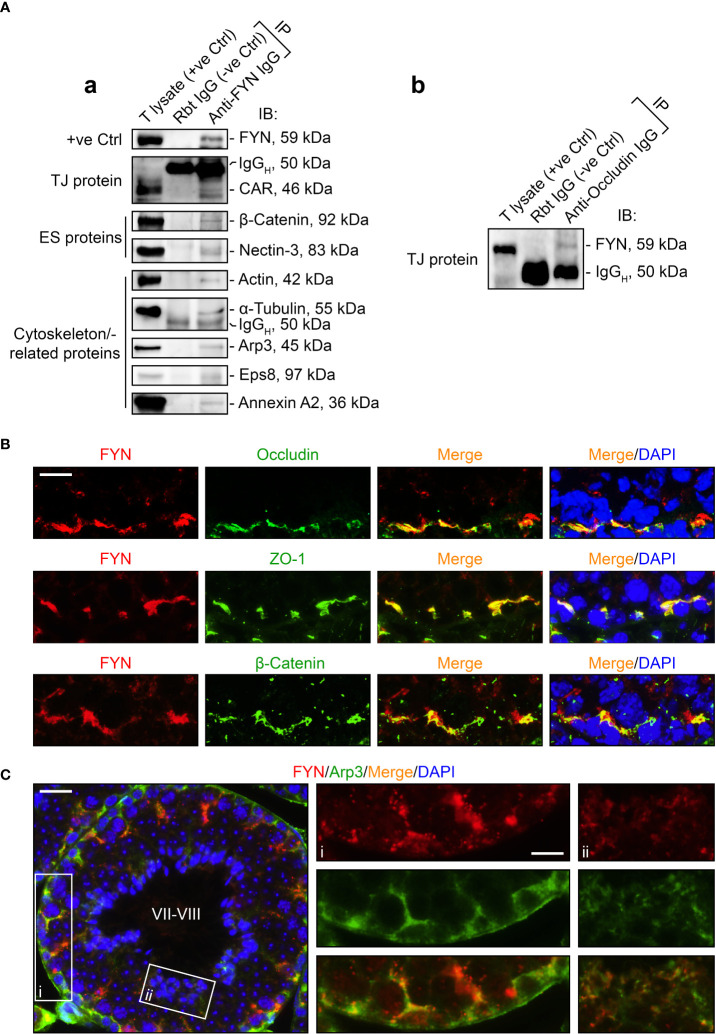
FYN is an integral component of the BTB and apical ES as demonstrated by Co-IP and dual-labeled IF assays. **(A)** Co-IP assays to determine the physical interactions between FYN and selected BTB/apical ES-associated proteins [including TJ proteins CAR **(A, a)** and occluding **(A, b)**, basal ES protein β-catenin, apical ES protein nectin-3, as well as cytoskeletal proteins actin, α-tubulin, Arp3, Eps8, and annexin A2] using mouse testis (T) lysates (~800 µg total protein/sample). A positive control (+ve Ctrl) was either testis lysate (30-50 µg protein/lane) without Co-IP, or the immunoprecipitated FYN was detected using its own antibody by immunoblotting **(A, a)**. For the negative control (-ve Ctrl), anti-FYN IgG was replaced with rabbit (Rbt) IgG. Several immunoblots show the heavy (IgG_H_) and light (IgG_L_) chains of IgG to better illustrate Co-IP results. **(B)** Dual-labeled IF staining to corroborate FYN localization at the BTB by its co-distribution with TJ proteins occludin and ZO-1, as well as basal ES protein β-Catenin in the testis. Scale bar, 10 μm. **(C)** FYN co-localization with actin binding protein Arp3, a key regulator of branched actin nucleation at the BTB and apical ES, further substantiated its presence at the BTB and apical ES (45). The stage of the seminiferous epithelium is indicated in Roman numerals. Scale bar, 16 μm. Boxed areas denoted with i and ii (showing FYN-Arp3 co-localization at the BTB and apical ES, respectively) are enlarged and presented to the right in the second and third columns, respectively (Scale bar, 8 μm).

As FYN expression pattern closely followed those of the BTB- and apical ES-associated proteins and actin-binding proteins (**Figure 2A**), we sought to identify any interaction or co-localization between FYN and these proteins to confirm its synergistic function at the BTB/ES sites. To investigate this, Co-IP experiments were carried out to determine whether FYN interacted structurally with TJ, ES, and the underlying cytoskeletal proteins (**Figure 3A**). Our choice of proteins was based on the fact that the antibodies were optimized for the experiments and the proteins were demonstrated to be elements of the BTB and/or apical ES (2, 34–36). As revealed by Co-IP results (**Figure 3A**), FYN structurally interacted with the TJ proteins CAR [also a component of the apical ES (38)] and occludin (**Figure 3A**, **a**, **b**); with the basal ES protein β-catenin; with the apical ES protein nectin-3; and with the cytoskeleton proteins actin and α-tubulin. Furthermore, FYN was found to be physically linked to the actin-binding proteins Arp3, Eps8, and annexin A2. These proteins are important actin regulators that have been shown to control the integrity of the BTB and the apical ES (36, 45–47). These findings suggest that FYN contributes to the actin cytoskeleton-based cell junctions, including TJ and basal ES at the BTB, as well as the apical ES at the Sertoli cell-spermatid interface. To expand on the Co-IP findings, tissue sections of adult mouse testes were immunostained with dual fluorescent dyes and analyzed. FYN and occludin were partially overlapped by IF microscopy when corresponding images were merged, supporting the Co-IP analysis (**Figure 3B**, upper panel). Co-localization of FYN and ZO-1 was yet another indication of FYN’s existence at the BTB site (**Figure 3B**, middle panel). IF showed the same result as Co-IP for partial co-localization of FYN with the basal ES protein β-catenin at the BTB in the seminiferous epithelium (**Figure 3B**, lower panel). The presence of FYN at the BTB and the apical ES was further substantiated by the high degree of overlapping it displayed with Arp3 (**Figure 3C**), a subunit of the Arp2/3 complex that disrupts the actin cytoskeleton at the BTB and apical ES by driving actin filaments into a branching network (45, 47). The close resemblance of FYN staining pattern to Arp3 suggests this protein kinase may modulate actin dynamics at the BTB and apical ES through interaction with the Arp2/3 complex.

### FYN expression decreases during CdCl_2_-induced disruption of cell adhesions and disorganization of the actin filament network in the testes

**Figure 4:**
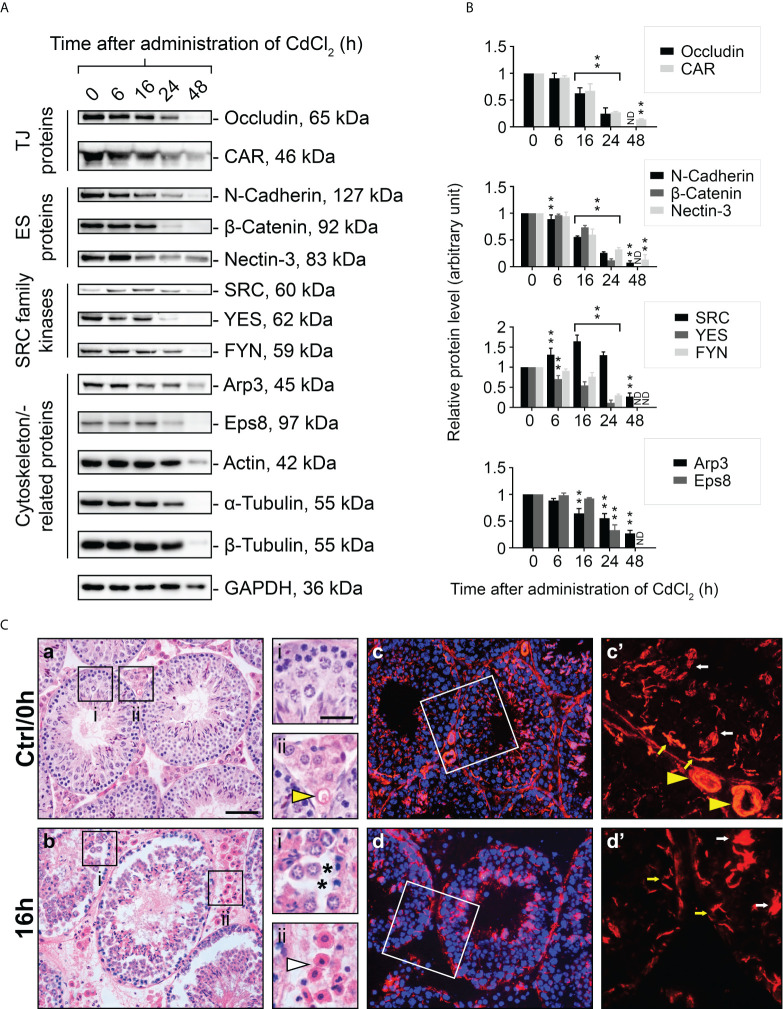
A study to assess changes in BTB/apical ES-associated protein levels, testis morphology, and F-actin distribution following CdCl_2_ administration. Immunoblotting experiments showing changes in levels of TJ, ES proteins, and SRC family kinases, as well as cytoskeletal proteins **(A)**. GAPDH was used as an internal control. Histograms **(B)** summarizing immunoblotting results. A normalization was performed by comparing each data point to its corresponding GAPDH data point and then to ‘0 h’, which was set to 1 arbitrarily. Each bar represents the mean ± S.D. of n=7-10 mice. **P<0.01 by one-way ANOVA with Dunnett’s test. ND, not detected. The histogram for actin, α- and β-tubulin is not shown. As part of the SRC family, SRC was up-regulated in testis following CdCl_2_ treatment, while YES and FYN was down-regulated. **(C)** Representative images of H&E staining of 5 μm-thick paraffin-embedded sections **(a, b)** and immunofluorescence staining of 7 μm-thick frozen sections **(c, d)** obtained from control (Ctrl/0h) and 16-hour CdCl_2_-treated mice (16h). DAPI was used to visualize nuclei **(c, d)**. The boxed areas (in **a, b, c**, and **d**) correspond to magnified images which are shown to the right of each low magnification image. Yellow arrowheads (Ctrl/0h, ii and c’) refer to intact blood vessels in the interstitial tissue of the testis. Asterisks (16h, i) indicate tears in the seminiferous epithelium after germ cells have detached. White arrowhead (16h, ii) points to outflow of blood cells indicating testicular hemorrhage. Yellow and white arrows **(c’, d’)** denote the expected locations of the BTB and apical ES, respectively. Bar (**a**, also applies to **b, c**, and **d**) = 25 μm; bar (i, also applies to ii, c’ and d’) = 10 μm.

The use of an *in vivo* cadmium treatment model allowed us to better understand the role of FYN in cell junctions between Sertoli-Sertoli and Sertoli-germ cells. A single i.p. injection of CdCl_2_ at a dose of 3 mg/kg b.w. has been shown to disrupt the BTB through the action on actin structures in Sertoli cells over a period of 7-16 hours and cause the sloughing of germ cells from the seminiferous epithelium, as well as the development of visible vascular damage in the testes (24–27). Upon treatment with CdCl_2_, the expression levels of all proteins examined declined overall, except SRC kinase. The steady-state expression level of SRC was elevated within 6 to 24 hours, but not that of YES or FYN, which belong to the same family of kinases as SRC (**Figures 4A, B**). It was consistent with previous reports that SRC expression level increased during germ cell detachment (24, 25). Almost all protein levels had diminished dramatically by 48 hours after CdCl_2_ exposure. By this point, the BTB was irreversibly disrupted and the seminiferous epithelium was nearly devoid of germ cells, thus most immunoreactivity of proteins in the testis had largely vanished. By immunoblotting, the protein level of FYN in the testes was steady between 0-16 hours following CdCl_2_ treatment, however, by 24 hour post-treatment, FYN level had declined significantly, and was no longer detectable by 48 hours (**Figures 4A, B**). Those results suggest that FYN may play an important role in the early sloughing of germ cells as well as the restructuring of cell junctions.

Using H&E staining, we examined the structural changes that occur in the seminiferous epithelium during CdCl_2_ treatment. The seminiferous epithelium was intact in the control (Ctrl/0h) group with respect to morphology and structure, and the interstitial blood vessels were clearly visible (**Figure 4C**, **a**). However, by 16 hours after CdCl_2_ treatment, the germ cells had peeled off, there were noticeable torn spaces in the epithelium, and blood cells had overflowed from the blood vessels and spread into the interstitial space (**Figure 4C**, **b**). Rhodamine phalloidin was used to visualize the actin cytoskeleton in the seminiferous epithelium. In the control group (**Figure 4C**, **c**, **c**’), actin filaments were distributed orderly at both the apical ES and the BTB sites, and the blood vessels were also uniformly arranged. However, after 16 hours of CdCl_2_ treatment (**Figure 4C**, **d**, **d**’), actin filaments were disorganized and clumped at the apical ES, and lost their orderly structure at the BTB. Within the interstitial spaces of the testes, the actin filament structures were severely reduced, and in some areas hardly visible.

### Arp3 binding to FYN and tyrosine phosphorylation are enhanced during CdCl_2_-induced junctional restructuring in the testes

**Figure 5:**
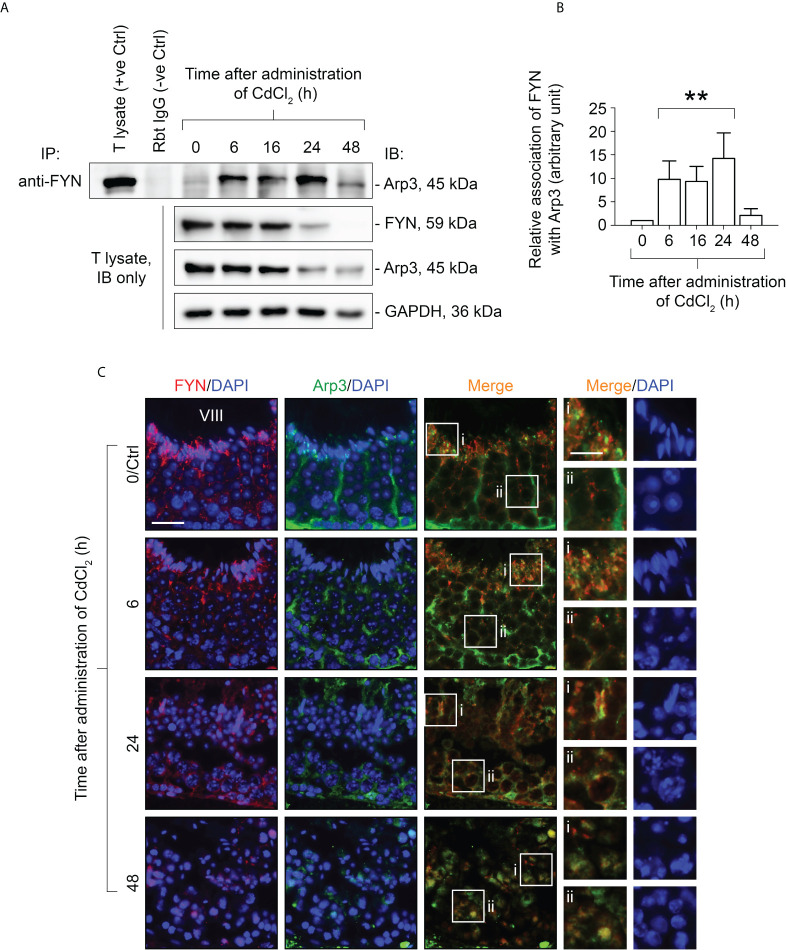
Co-immunoprecipitation (Co-IP) study to evaluate changes in protein-protein interactions between FYN and Arp3 during CdCl_2_-induced junctional restructuring **(A)**. For Co-IP experiments, ~800 µg testis (T) lysate from CdCl_2_-untreated and treated mice was incubated with anti-FYN IgG, followed by immunoblotting (IB) with an Arp3 antibody (**Table 1**). Also shown was the change in FYN and Arp3 protein levels in response to CdCl_2_ administration. GAPDH served as an internal control. **(B)** A histogram summarizing the results of Co-IP. FYN association with Arp3 at 0 hour was arbitrarily set at 1. Each bar represents the mean ± S.D. of three independent experiments. **P<0.01 by one-way ANOVA with Dunnett’s test. **(C)** Dual-labeled IF experiments to evaluate how FYN and Arp3 co-localization changed in the seminiferous epithelium following CdCl_2_ administration. As of time 0/Ctrl, the epithelial cycle of the seminiferous tubule was clearly identified as stage VIII, which is given in Roman numerals. After 24 hours, the seminiferous epithelium had collapsed and it was impossible to determine its stage. The boxes denoted with i or ii in the third column (Merge) are magnified in the fourth column and their corresponding DAPI shown on the right (fifth column). Bar = 20 μm (first column, also applies to second and third columns); bar (magnified views in the fourth and fifth columns) = 10 μm.

**Figure 6:**
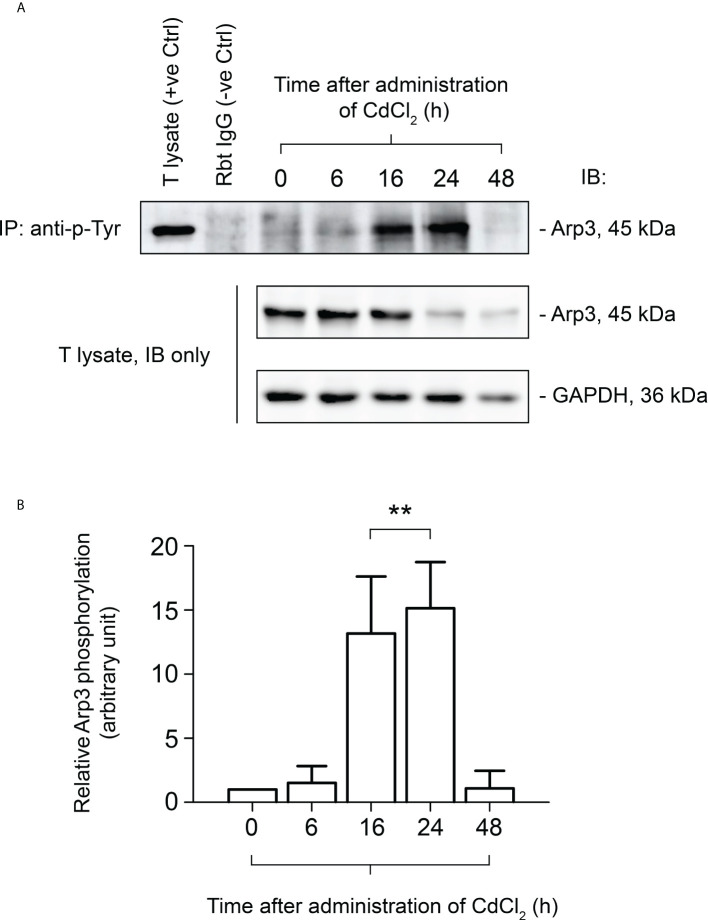
Co-immunoprecipitation (Co-IP) study to evaluate the tyrosine phosphorylation status of Arp3 during CdCl_2_-induced junctional restructuring. For Co-IP experiments **(A)**, ~800 µg testis (T) lysate from CdCl_2_-untreated and treated mice was incubated with anti-phosphotyrosine (p-Tyr) antibody, followed by immunoblotting (IB) with an Arp3 antibody (**Table 1**). The level of Arp3 in response to CdCl_2_ administration was also shown. GAPDH served as an internal control. **(B)** A histogram summarizing the results of Co-IP. The tyrosine-phosphorylated Arp3 level at 0 hour was arbitrarily set at 1. Each bar represents the mean ± S.D. of three independent experiments. **P<0.01 by one-way ANOVA with Dunnett’s test.

It has been demonstrated that CdCl_2_ disrupts the BTB by fragmenting actin microfilament bundles in Sertoli cells (27), and based on the above observations, it is reasonable to suggest an important role for FYN in the signaling process of microfilament fragmentation. We expect that this is achieved through its interaction with Arp3 at the cell junctions at the Sertoli-Sertoli and Sertoli-germ cell interfaces. As further experimental proof of this hypothesis and clarification of FYN’s role in CdCl_2_-induced cell junction disruption, we used the Co-IP technique to examine changes in the interaction between FYN and Arp3 following CdCl_2_ treatment (**Figures 5A, B**). According to the results of the Co-IP experiment, at time 0, the level of interaction between FYN and Arp3 was still low, but it increased between hours 6-24, and then dropped at 48 hours. The drop following 48 hours can be explained by the destruction of most of the seminiferous epithelium. It is interesting to note that FYN-Arp3 interactions persisted beyond 24 hours even after levels of FYN and Arp3 in testes began to decline after 16 hours of CdCl_2_ treatment (**Figures 4A, B**, **5A**), hence dual-labeled IF experiments were performed to evaluate how their co-localization changed in the seminiferous epithelium (**Figure 5C**). During time 0, FYN and Arp3 were partially co-localized at the apical ES and Sertoli/germ cell adhesion; however, six hours after CdCl_2_ treatment, FYN and Arp3 staining patterns were already perturbed and fragmented in the seminiferous epithelium, and their localizations at the apical ES and Sertoli/germ cell adhesion were more diffuse and overlapped. After 24 hours, BTB and apical ES were no longer prominent, and FYN and Arp3 staining also had a lack of appropriate presence at the sites, but they formed obvious clusters nonetheless. Germ cells were greatly depleted from the epithelium, causing even further epithelial tears. While FYN and Arp3 staining were greatly reduced over 48 hours, their association with messy germ cells continued to overlap. In this way, the dual-labeled IF experiments confirmed the results of the Co-IP experiment, revealing increased binding of FYN and Arp3 at the Sertoli-germ cell interface, and showing that FYN and Arp3 interact to facilitate germ cell expulsion. According to our hypothesis, the increased interaction between FYN and Arp3 may result in a change in the level of Arp3 tyrosine phosphorylation, thereby altering Arp3’s ability to regulate the polymerization of branched actin filaments at the apical ES and the BTB, leading to germ cell detachment from the epithelium and disintegration of the BTB. Co-IP was used again to measure tyrosine phosphorylation changes of Arp3 after CdCl_2_ treatment (**Figure 6**). Compared to time 0, the Arp3 phosphorylation level did not change significantly after 6 hours of CdCl_2_ treatment. However, it rose dramatically at 16-24 hours, and then decreased at 48 hours. The data showed that Arp3 played an essential role in the 16-24 hours germ cell departure as well as the large-scale destruction of the seminiferous epithelium. The fact that Arp3 and FYN showed a sharply increased interaction at 6-24 hours suggests that tyrosine phosphorylation of Arp3 may be partly a consequence of FYN-dependent catalysis, and that the interaction precedes the phosphorylation of Arp3 by FYN (**Figure 5** versus **Figure 6**). Considering that the steady-state levels of FYN and Arp3 decreased after CdCl_2_ treatment (**Figures 4**, **5**), the transient surge in FYN-Arp3 interaction and Arp3 phosphorylation status was unlikely to be the result of a superposition of steady-state expression levels of the two, or by a loss of germ cells that altered cell ratios. As a further point to note, at 24 hours, FYN and Arp3 protein levels dropped sharply, but their interaction and tyrosine phosphorylation level of Arp3 remained at a level comparable to 16 hours. It can be explained by the fact that when using lysates with the same total protein (~800 µg), Co-IP experiments at 24 hours show rather amplified results compared to 16 hours.

## Discussion

### FYN modulates underlying Sertoli cell cytoskeleton(s) in the mouse testes

In this study, we reported a relatively stable and high expression of FYN at the Sertoli-Sertoli cell and Sertoli cell-spermatid adhesion in the adult mouse testes. A radial pattern of FYN distribution was observed in the Sertoli cell stalk (**Figure 1**), suggestive of a possible intimate association between FYN and other Sertoli cell cytoskeleton(s) in addition to the well-known presence of FYN at the actin-based ES (17), that is, FYN may also associate with microtubules and/or vimentin intermediate filaments, which stain along the Sertoli cell stalk as well (33, 48–50). In prior studies, actin and microtubule cytoskeletons were shown to be involved in germ cell transport, during which microtubule tracks are used to convey spermatids and are closely associated with apical ES. As spermatids develop, they travel “down and up” the seminiferous epithelium before reaching the apical regions for spermiation. Apical ES is thought to move along nearby microtubules by engaging either plus or minus end motor proteins (49, 51–56). Residual bodies are also transported by the microtubule-based cytoskeleton from the apex to the base of the Sertoli cell cytoplasm. Also, FYN was detected in the tubule lumen surrounding residual bodies (**Figure 1**). It remains to be confirmed whether FYN is present at vimentin intermediate filament-based desmosomes and actin-based gap junctions, which would suggest a role for FYN at the interface of Sertoli cells and pre-step 8 germ cells, i.e., in early-stage germ cell transport. Consequently, a partial confirmation was provided by the finding that FYN binds to α-tubulin (**Figure 3A**). The study indicates that FYN may be involved in germ cell transport by carrying out activities such as assembling and disassembling Sertoli-germ cell junctions, removing apical ES and cytoplasmic residues of spermatozoa, and phagocytosing residual bodies in the seminiferous epithelium (33, 57, 58). Further studies on these topics may enable us to better understand how FYN contributes to immune barrier maintenance and germ cell transport across the epithelium.

### Can FYN have an effect on recruitment and distribution of the BTB and apical ES components?

Modulation of the phosphorylation status of BTB and apical ES components enables rapid assembly and disassembly of cell junctions in response to changes in the surrounding environment, such as movement of component proteins from the Sertoli-Sertoli cell interface to the cytosol to achieve the effect of dismantling cell junctions (2, 6). During postnatal testicular development (**Figure 2**) and CdCl_2_-induced junctional restructuring (**Figure 4**), FYN was found to closely follow the expression pattern of the BTB and apical ES-related proteins, specifically the TJ and basal ES (such as the occludin–ZO-1 and N-cadherin–β-catenin protein complexes at the BTB) and actin-binding proteins, such as Arp3, Eps8, and annexin A2. This suggests FYN may work in concert with these proteins, possibly modulating their activity and/or localization through FYN-dependent tyrosine phosphorylation (2, 12). Co-IP analysis has confirmed the association of FYN with these proteins (**Figure 3**). As the hypothetical scenario suggests, increased interaction between FYN and Arp3 preceded increased phosphorylation of Arp3 by FYN (**Figures 5**, **6**). Given that FYN is dually palmitoylated and directly targets the plasma membrane (12, 15), it may be able to recruit Arp3 to the juxtamembrane region, where it can then phosphorylate and activate it.

### An alternative explanation for the mutual replacement of SRC, YES, and FYN during spermatogenesis

It has been proposed that SFK family members play overlapping roles in multiple cellular events, and most of the functions performed by members of the SFK family other than SRC can be replaced by SRC. Specifically, triple knockouts of SRC, YES, and FYN result in embryonic lethality, double knockouts of SRC and YES, or SRC and FYN, result in perinatal lethality, and a single knockout of SRC causes postnatal lethality or infertility, but a single knockout of YES or FYN is viable and fertile (11–13). These findings seem to emphasize the redundant roles of SRC and YES, or SRC and FYN. However, previous studies have indicated that SRC and YES play different roles in endocytic vesicle-mediated trafficking events at the Sertoli cell BTB. SRC is involved in degradation of integral membrane proteins and hence cell junction disassembly, while YES is involved in recycling and/or transcytosis of integral membrane proteins and maintenance of the cell junctions (12, 13, 16).

In this study, FYN was found to be physically linked to the actin-binding proteins Arp3 and Eps8 (**Figure 3**). Interestingly, Arp3 and Eps8, two important actin regulators, exert antagonistic roles in maintaining BTB and apical ES integrity: Arp3, a subunit of the Arp2/3 complex, destabilizes the actin cytoskeleton at the BTB and apical ES by driving polymerization of branched actin filaments. Eps8, on the other hand, maintains the integrity of cell-cell junctions in the testis by capping and bundling actin filaments (11, 45–47). In contrast, YES has been shown to structurally interact with Eps8, but not with Arp3 (30). Based on these findings, it is possible that unlike YES, which has a single role that is to reinforce the integrity of the BTB and apical ES, FYN has a dual or alternate role, such as when it interacts with Eps8, it positively correlates with the junctional integrity, and when it interacts with Arp3, the reverse occurs.

A previous study reported that although male FYN knockout mice were fertile, a significant reduction in testis weight and degeneration of germ cells were observed at 3 and 4 weeks of age, the time of BTB *de novo* formation in the juvenile testes. There were abnormalities in the actin filament layers of the ES in FYN mutant testes. By adulthood, however, the mice appeared to be normal and fertile again (17). As can be seen from the analysis above, there is a possibility that FYN itself can both be “good” and “evil” for the integrity of the cell junctions, and its action can be tailored to specific situations. During postnatal development into adulthood, its “good” and its “evil” can be replaced or rescued by YES and SRC, respectively. Because reproduction is crucial to the continuation of all life forms, this may simply be a stress response in the testis. It does not mean, however, that FYN is dispensable.

### Role of FYN at Sertoli-Sertoli and Sertoli-germ cell junctions in rodent testes: A model

**Figure 7:**
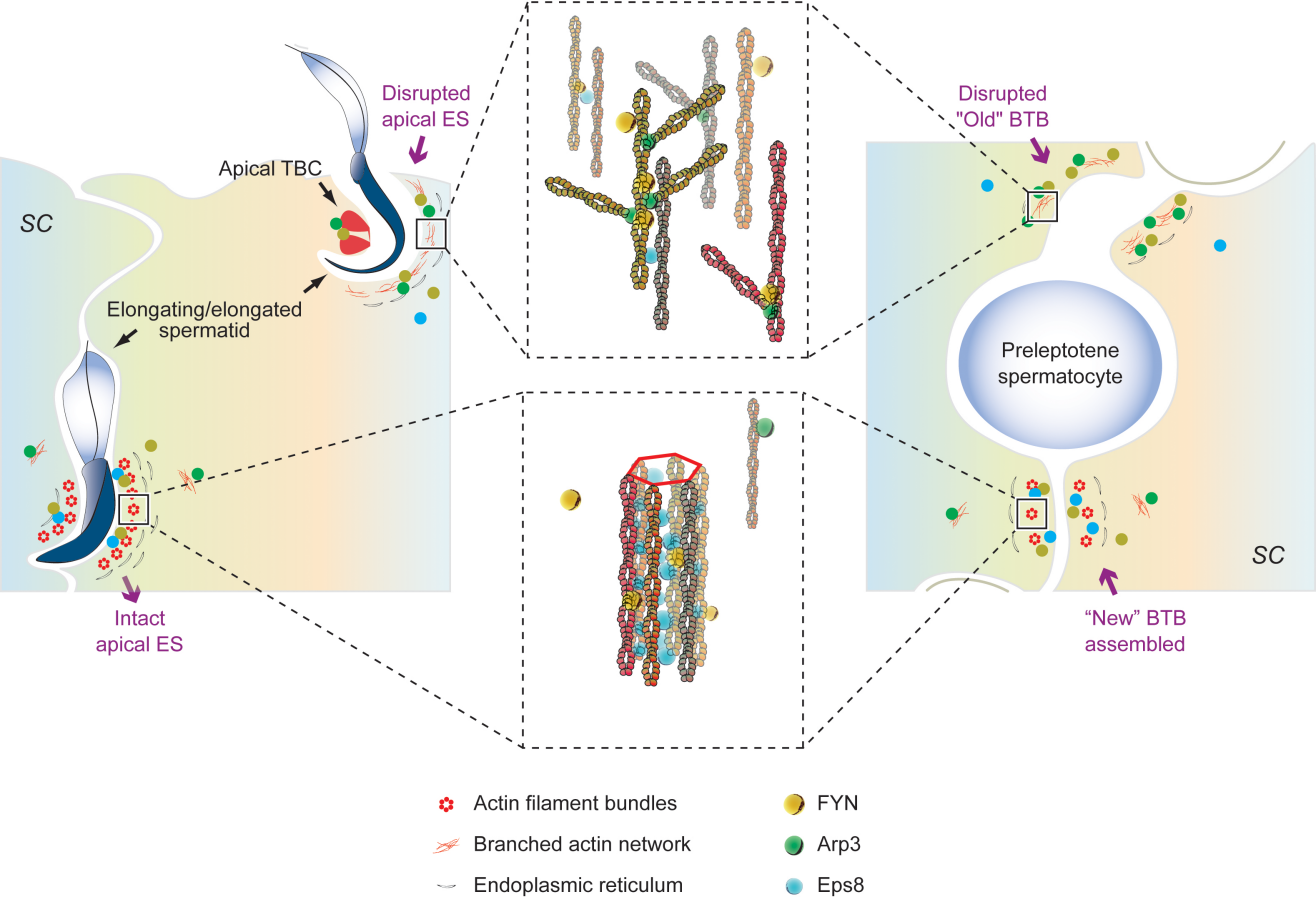
An illustration of our proposed working hypothesis in the rodent testes by which FYN, Arp3, and other actin-associated proteins, such as Eps8 and Annexin A2, regulate the apical ES and the BTB to accommodate the transport of spermatids in the seminiferous epithelium, and preleptotene spermatocytes across the BTB from the basal to adluminal compartments, respectively. Bottom left panel: the intact apical ES between elongating spermatids and Sertoli cells is maintained through a network of actin filament bundles. This network is created by the actin barbed end capping and bundling protein Eps8, which binds FYN as well. FYN and Eps8 may function together to promote the assembly or integrity of the apical ES (bottom middle panel), which has yet to be clarified. A change in localization and/or distribution of the actin regulatory proteins, Arp3 and Eps8, is observed in stages VII-VIII tubules as elongated spermatids (e.g., step 19 and 16 spermatids in rats and mice, respectively) are about to leave, or after exposure to toxicants such as CdCl_2_. The actin nucleation and branching protein Arp3 interacts with FYN and likely gets phosphorylated and activated. Arp3 promotes the de-bunching of the well-organized F-actin bundles into branched configurations, leading to microfilament truncation and retrieval from near the cell surface. In this way, the apical ES is disrupted, rendering it ineffective for supporting spermatid adhesion to Sertoli cells (up left and up middle panels), resulting in spermatid departures at the right time (at spermiation) or prematurely (following CdCl_2_ administration). Actin-rich apical tubulobulbar complexes (apical TBCs) associated with late-stage spermatids likely act as a giant endocytic vesicle-based protein trafficking device prior to spermiation, in order to remove cytoplasm from late spermatids (i.e., residual bodies) where Arp3, annexin A2, and FYN are found (36, 45) (upper left panel). At the BTB (right panel), the same thing happens. A network of actin filament bundles between adjacent Sertoli cells maintains BTB integrity, which is supported by Eps8; FYN may cooperate with Eps8 to achieve this result (bottom right panel). In stage VIII or CdCl_2_-induced BTB restructuring, Arp3 converts F-actin to branched network, disrupting the BTB (right panel). As part of normal spermatogenesis, the “new” BTB is assembled before the “old” BTB is disassembled, so the integrity of the immunological barrier can be maintained. FYN can regulate the actin regulatory proteins (Arp3, Eps8, and annexin A2) and act as a switch or signal hub in response to signals of junctional restructuring in the testis. This model thus depicts the crucial physiological role FYN plays at the apical ES (left panel) and BTB (right panel), respectively, during seminiferous epithelial cycle.

We have shown that FYN, a non-receptor tyrosine kinase, is an important regulatory protein possibly involved in the maintenance of BTB integrity, spermatid adhesion, and spermiation *in vivo*. While other signaling pathways still need to be identified, FYN effects on BTB and apical ES appear to be mediated by phosphorylation and/or recruitment of Arp3. We propose a hypothetical model in the rodent testes whereby FYN, Arp3, and other actin regulatory proteins, such as Eps8 and Annexin A2, modulate the apical ES and the BTB to accommodate the transport of spermatids in the seminiferous epithelium, and preleptotene spermatocytes across the BTB from the basal to adluminal compartments, respectively (**Figure 7**). The intact apical ES is maintained by a network of actin filament bundles. In interaction with FYN, the actin nucleation and branching protein Arp3 likely gets phosphorylated and activated. Arp3 promotes the de-bunching of well-organized F-actin bundles into branched configurations, resulting in microfilament truncation and retrieval from near the surface of the cell. By disrupting the apical ES, spermatid adhesion to Sertoli cells is impaired, causing spermatids to depart either at the appropriate time (at spermiation) or prematurely (after CdCl_2_ administration). The BTB follows the same pattern. Thus, this model illustrates the critical physiological role FYN has at the apical ES and BTB during the seminiferous epithelial cycle. Additionally, a number of viruses have been shown to disassemble TJ or AJ in infected endothelial/epithelial cells that can be blocked by SFK inhibitors such as dasatinib, PP1, and PP2 (20–22). FYN was found to respond rapidly to virus exposure, being specifically necessary for virus internalization in Caco-2 cells, a model of intestinal epithelial barrier (59). As shown in our studies, FYN and Arp3 function together to “open” the BTB, suggesting a mechanism for viruses to enter the testes through the BTB.

## Data availability statement

The original contributions presented in the study are included in the article/supplementary material. Further inquiries can be directed to the corresponding author.

## Ethics statement

The animal study was reviewed and approved by Institutional Animal Care and Use Committee of Hangzhou Medical College (Zhejiang Academy of Medical Sciences).

## Author contributions

XX supervised the study. YY, MY, and JZ performed experiments. DZ, QL, and YN contributed new reagents/analytic tools; YY, MY, and XX performed data analysis and generated the figures and table; XX wrote the manuscript with input from YY and MY. All authors contributed to the article and approved the submitted version.

## Funding

This work was supported by grants from the National Natural Science Foundation of China (31371176 and U20A20133), Zhejiang Provincial Natural Science Foundation of China (LY21H040005), Zhejiang Provincial Department of Education (Y202045395), Health Commission of Zhejiang Province (2020RC052), Hangzhou Medical College 2021 Institutional Special Fund (YS2021007), Applied Research Project on Public Welfare Technology of Zhejiang Province (LGC19H280008).

## Conflict of interest

The authors declare that the research was conducted in the absence of any commercial or financial relationships that could be construed as a potential conflict of interest.

## Publisher’s note

All claims expressed in this article are solely those of the authors and do not necessarily represent those of their affiliated organizations, or those of the publisher, the editors and the reviewers. Any product that may be evaluated in this article, or claim that may be made by its manufacturer, is not guaranteed or endorsed by the publisher.
